# Epulis

**Published:** 1895-11

**Authors:** A. H. Beers

**Affiliations:** Cookshire, Que.


ARTICLE V.

### EPULIS.

BY A. H. BEERS, M. D., D. D. S., L. D. S., COOKSHIRE, QUE.

This diseased condition is one not foreign to the practice of dentistry, as the tumor is frequently met with in ordinary practice. I intend to give a brief description of this tumor, and also report a case that I had the opportunity of observing some time ago under the supervision of several distinguished surgeons of Montreal. The term

"epulis" is applied to various tumors of the gums. In reality they are not connected with the gums, but with the periosteum of the alveolar process, especially that part intimately connected with the teeth.

Two forms of this tumor are usually described: simple, or the fibrous variety, and the form that contains myeloid elements and is considered as a malignant type.

The former is the more common, and is seen as an enlargement of the natural fibrous tissue of the gum. It is covered with mucous membrane continuous with that of the gum, but from various mechanical causes—such as the teeth, tongue, etc—are liable to become inflamed and lacerated, and thus stimulate a malignant growth. Occasionally this growth presents ossified parts, and in this respect differs from the myeloid variety, which never ossifies. Spiculæ of bone may be prolongated from the maxilla into the fibruous variety. It may grow between teeth and gradually force them apart as it increases in size, and produce great disfigurement. The myeloid form is much more vascular and increases in size more rapidly. It frequently exhibits itself as a large fungating growth, and is always more serious in nature and more apt to occur after removal.

The treatment of these two forms amount to about the same thing—that is, entire removal of tissues that surround the growth. Its occasional appearance in apparently edentulous jaws will be found, on close examination to be due to overlooked roots of teeth, as a rule. Its occurrence is dependent upon the presence of teeth. Nothing short of the entire removal of the tooth or teeth and bone that is contiguous will ever effect a cure. I lately saw a case that had been treated by a medical gentlemen. He merely snipped the pedicle six times, and it always came again. The cause was an irritating left lower cuspid, and the growth extended from the right lower incisor to the second molar. The growth was indented by the teeth, and consequently ulcerated. The breath was extremely fetid, and signs of constitutional disturbance present. The growth was, roughly speaking, the size of a hen's egg, but more flattened, of course. It was simply fibrous and of slow growth, but was ulcerated from indentation of the upper teeth. The continual absorption of pus would naturally account for constitutional disturbances.

I saw a case in a young girl, aged 17, which I will try to report.

January 20th.—Came complaining of swelling on inner right side of alveolar process of upper jaw and in region of molar teeth; frontal headache and general weakness. Patient first noticed swelling seven months ago, and supposed it to be a "gum-boil." The swelling was lanced by a dentist, and after a few days was "removed." It recurred in a month. Two months later it was removed with a dens sapientia, which had partly cut through the gum. Soon after this the tumor reappeared, and increased to the size in which I saw it—that of a walnut. Movements of the tongue and eating caused slight bleeding. Her father and mother are alive and healthy. Twelve brothers and sisters died in infancy; two brothers and one sister alive and well. One uncle has benign growth on chin. Grandmother dying with cancer of rectum. The patient is a tall and very well nourished girl, and is otherwise healthy, as was proved from physical examination. The right tonsil and pharynx and surrounding mucous membrane are injected; growth bleeds readily on slightest irritation.

January 25th.—Patient etherized. Mouth forced open and kept so with gag. Second right upper molar removed, and most of the growth which was adhering to the dental periosteum came away with the tooth. Remainder of growth and part of alveolar border of jaw cut away with forceps.

January 26th.—Tissues in neighborhood considerably swollen. A mouth-wash (warm) of boracic acid given.

January 27th.—Swelling considerably reduced. Diet, beef-tea and milk.

January 30th.—Patient discharged, with wound in a healthy condition.

February 18th.—Patient was seen again, with recurrence of growth, probably due to incomplete removal and originating from some part left behind in the former operation. Under ether, the first upper right molar was removed and alveolar process removed with bone-forceps. Roots of tooth were very divergent, and much difficulty was experienced in removing it entire. The growth was examined microscopically, and found to be a simple fibrous tissue growth. The patient, after six months, has had no recurrence.—*Dominion Journal.*

